# Depression in military wives: the particularities of an understudied population

**DOI:** 10.1192/j.eurpsy.2025.2395

**Published:** 2025-08-26

**Authors:** S. Boughdadi, M. A. Laffinti

**Affiliations:** 1 Psychiatry, Mohammed VI university hospital; 2 Psychiatry, Avicenne military hospital, Marrakech, Morocco

## Abstract

**Introduction:**

Military spouses often experience separation from their husbands and the risks associated with their deployment in difficult environments. Multiple studies have focused on depression in service members, but little has been done to understand how demographic and military-specific factors affect their wives.

**Objectives:**

The goal of our study is to describe the demographic and military characteristics specific to women with a depressive disorder diagnosis, and are or were married to service men. We also aim to identify possible correlations between the beginning and evolution of the spousal depression and those characteristics.

**Methods:**

This was a retrospective descriptive study. Data was obtained from the medical files of military spouses followed at the psychiatry department of the military hospital in Marrakech between May 2022 and August 2023. Demographic and military-specific information unavailable in the files was collected through phone calls. Inclusion criteria were: a DSM-5 depressive disorder diagnosis, presence at follow-ups, being or having been married to a service man, an available functioning phone number. The statistical analysis was done on the software tool Jamovi® version 2.3.28.

**Results:**

All of the 25 women who were included in our study were diagnosed with major depressive disorder (MDD). Demographic factors related to MDD were noted in most spouses: 64% had a low educational level, 84% were housewives. Concerning the military factors related to service men: we observed that in the 24% of cases where the husband was deployed or lived apart from his family, there was either no or little improvement of depressive symptoms under treatment for the wife. Only 4% of service men had been diagnosed with a psychiatric disorder (MDD) at the time of our study. 44% of our patients reported conflicts with their husbands, out of these men 54% were still active in military forces. 54% of the patients with marital conflicts reported little to no improvement of their symptoms. 20% of women were victims of domestic violence.

**Conclusions:**

Our findings were able to show a high percentage of demographic risk factors for MDD in the studied population. They also imply a correlation between the husband’s deployment and the higher family responsibility reported by the wife in these cases, and a lack of improvement of the depressive symptoms under treatment. Although previous studies reported a correlation between the husband’s history of psychiatric disorder and the wife’s depression, we didn’t reach the same conclusion, this could be limited by the size of our study sample, as well as possibly underdiagnosed disorders in service men. These findings suggest the need for a specific approach for this population, as well as additional support services to help prevent and improve the treatment of depressive disorders in military wives.

**Disclosure of Interest:**

None Declared

